# Case Report: long-term calcaemia management in a dog after thyroidectomy and parathyroidectomy

**DOI:** 10.3389/fvets.2026.1663570

**Published:** 2026-02-19

**Authors:** Alessio Ruggiero, Camilla Sangiuliano, Monica Isabella Cutrignelli, Luigi Navas, Domenico Bergero

**Affiliations:** 1 Department of Veterinary Medicine and Animal Production, University of Napoli Federico II, Naples, Italy; 2 Department of Veterinary Science of the University of Turin, Grugliasco, Italy

**Keywords:** calcitriol, calcium, homemade diet, hypocalcaemia, vitamin D

## Abstract

This report describes a canine case of severe hypocalcaemia following the surgical removal of a thyroid lobe and the ipsilateral parathyroid glands for thyroid carcinoma. After initial stabilisation with intravenous calcium gluconate, the dog was initially managed with a commercial diet, and subsequently with a homemade diet, which provided 3.72 and 3.30 g/1,000 kcal ME of calcium, 376 and 295 IU of vitamin D3, and 2.48 and 1.50 g/1,000 kcal metabolisable energy (ME) of phosphorus, respectively. Both nutritional plans were supplemented with calcium carbonate (200 mg/day) and calcitriol (9.84 IU/day) to maintain normocalcaemia. A tailored, homemade diet was formulated to address the onset of hyporexia. The patient maintained stable serum calcium, phosphorus, and total 25(OH) vitamin D concentrations over a mid-term follow-up period of 6 months.

## Introduction

Blood calcium levels are tightly regulated by hormonal pathways involving multiple organs, including the thyroid, the parathyroid, the kidneys, the bone, and the liver, and three principal hormones: calcitonin, parathyroid hormone (PTH), and vitamin D (1). In particular, PTH increases calcium mobilisation from the bones and enhances renal reabsorption, while also stimulating the activation of vitamin D3 (1,25-dihydroxycholecalciferol), which in turn increases intestinal absorption of calcium and phosphorus. When blood calcium concentrations are elevated, negative feedback on the parathyroid glands suppresses PTH secretion. Calcitonin, secreted by the C cells of the thyroid gland in response to increased levels of ionised calcium (iCa), counteracts the effects of PTH by reducing serum calcium concentrations through the promotion of calcium deposition in bone (1).

Disorders of calcium homeostasis can arise from a diverse range of aetiologies, including nutritional deficiencies in calcium or vitamin D, endocrine dysfunctions, chronic kidney disease, and malignancies associated with skeletal metastasis or paraneoplastic syndromes (2). In different types of tumours, the expression of PTH-related protein, which exhibits an effect similar to PTH and is produced independently of calcium levels, has been described (3). Scruggs et al. (4) have reported that thyroid carcinoma can cause paraneoplastic hypercalcaemia in dogs. Chronic hypercalcaemia can result in damage to certain tissues and organs, such as the liver and kidneys (5–7).

Thyroid neoplasia can be treated by surgical excision or radiotherapy. During surgery, the parathyroid glands may also be inadvertently removed, particularly when macroscopic differentiation between thyroid and parathyroid tissue is not possible (8). Consequently, calcium metabolism is deregulated, and serum calcium levels may drastically decrease. Hypocalcaemia may be transient or persistent, with signs such as tremors, ataxia, and cardiac arrhythmia, which can vary in severity and may require admission to an intensive care unit (ICU). At this stage, it is crucial to rapidly restore the blood calcium levels (9). Although nutritional intervention is often recommended, its efficacy in long-term management has not yet been clearly demonstrated. Current literature on paraneoplastic hypercalcaemia primarily focuses on pharmacological management (8). When an alternative treatment is chosen and the parathyroid glands are affected, the long-term management of calcium, phosphorus, and vitamin D intake should be carefully adjusted according to serum levels.

This clinical case study focuses on the nutritional management of a dog following thyroidectomy and parathyroidectomy.

## Case description

**Figure 1:**
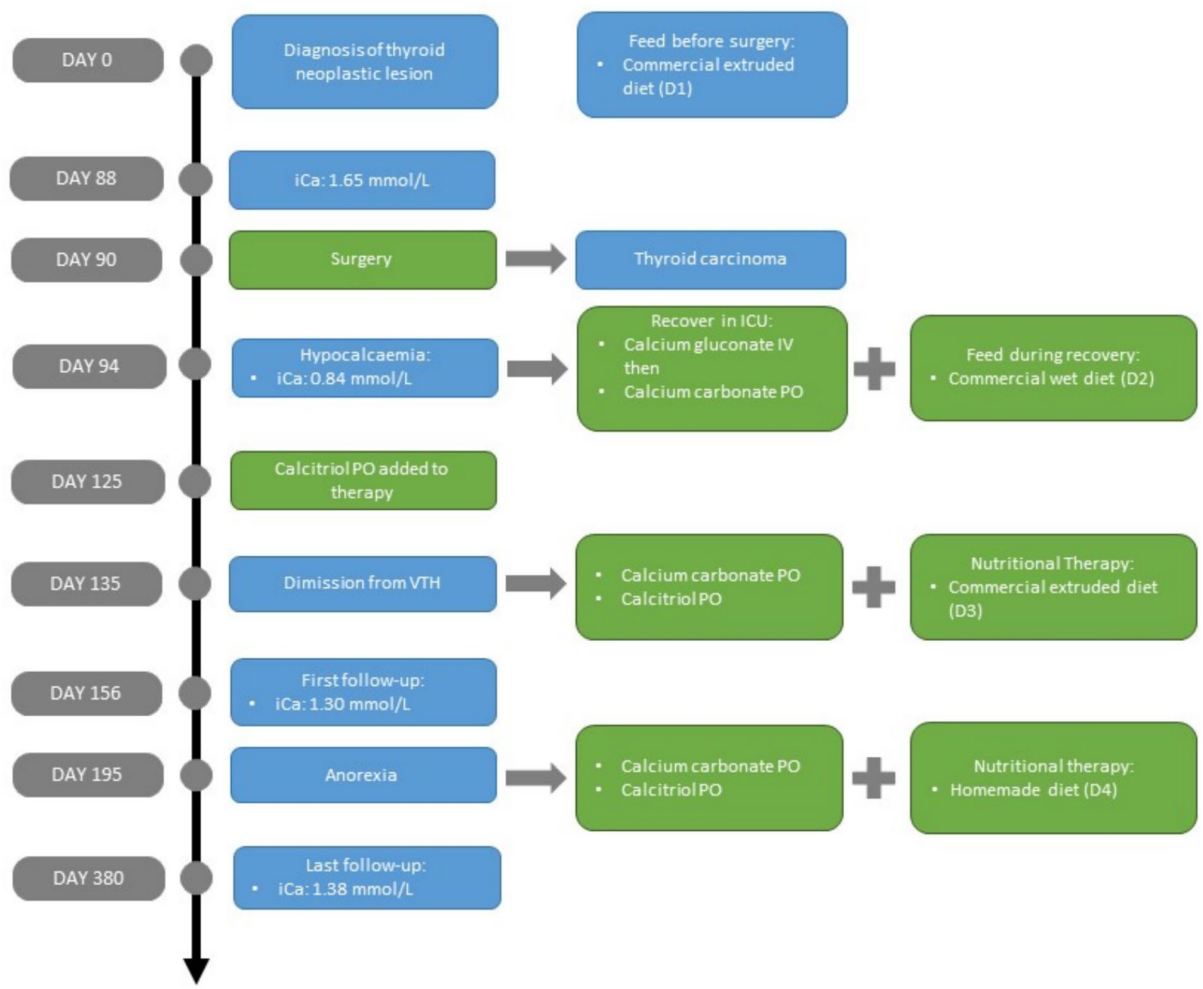
Timeline of the clinical follow-up of a canine patient focusing on hypocalcaemia control after parathyroidectomy. The most important aspects of the case (diagnosis, signs, and symptoms) are highlighted in blue, while treatment changes are highlighted in green. For information about diet and supplement characteristics, see Table 1: Diet 1, Diet 2, Diet 3, and Diet 4. iCa, ionised calcium; ICU, intensive care unit; IV, intravenous; PO, *per os*.

An 11-year-old spayed female Poodle dog was referred to the Veterinary Teaching Hospital (VTH) at the University of Naples Federico II in June 2023 (day 0) (Figure 1). The patient’s medical history included chronic hypercalcaemia, gallstones, and urinary bladder stones. In particular, approximately 6 months prior to day 0, the dog exhibited signs of urinary obstruction and underwent surgical intervention at another veterinary clinic, with no prior attempt at medical dissolution. On that occasion, hypercalcaemia was detected for the first time. However, the clinicians did not request an analysis of the urolith composition, even though hypercalcaemia predisposes to calcium-containing uroliths such as calcium phosphate or calcium oxalate (10). At the subsequent clinical evaluation (day 0), the dog weighed 8.2 kg, with a body condition score of 7/9 (BCS, 9-point scale) (11) and a normal muscle condition score (MCS) (12). The dog was fed a commercial extruded diet formulated to reduce calcium oxalate urolith formation (diet 1 [D1]) containing, according to the label, 1.58 g/1000 kcal of metabolisable energy (ME) of calcium, 446.66 IU/1000 kcal ME of vitamin D, and a phosphorus content (0.94 g/1000 kcal ME) slightly below the recommended level by Fédération Européenne de l’Industrie des Aliments pour Animaux de Compagnie (FEDIAF) (13) (Table 1). To avoid differences between manufacturers, the ME values for each diet were calculated according to National Research Council (NRC) based on crude fibre content (14). Physical examination revealed tachypnoea without mucosal abnormalities. All complete blood count parameters and the main biochemical biomarkers, including phosphate, magnesium, urea, creatinine, triglycerides, cholesterol, alanine aminotransferase, gamma-glutamyl transferase, total proteins, and albumin, were within the physiological range. However, alterations in the following parameters were observed: total serum calcium of 15.4 mg/dL [reference range (RR) 7.94–12.02] and alkaline phosphatase (ALP) of >1,000 IU/L (RR: 12–134). Palpation of the neck revealed a single round nodule corresponding to the left thyroid lobe (measuring approximately 12 mm in diameter) with an elastic consistency. As a consequence, a total body computed tomography (CT) scan with a contrast medium examination was performed on day 4 to evaluate the cause of hypercalcaemia. The CT scan revealed enlargement of the left thyroid lobe (maximum diameter: 14 mm), which appeared globular and hypodense before contrast injection, with intense enhancement after injection; the corresponding lymph node was also enlarged. In addition, gallstones, renal cortical microcysts, bilateral renal calcifications, and bladder stones were noted.

**Table 1:** Chemical composition and ingredients of the diets consumed by the described patient.

Nutrient (unit/1000 kcal of ME)	FEDIAF^1^	Diet 1	Diet 2	Diet 3	Diet 4
Time (days)		0–93	94–134	135–194	195–380
Moisture content (g)	-	23.64	252.45	22.39	254.10
Crude protein (g)	45.00	49.92	45.00	67.16	68.73
Fat (g)	13.75	49.92	29.74	34.82	32.05
Crude fibre (g)	-	9.72	11.41	6.21	2.78
Crude ash (g)	-	14.98	7.61	16.17	8.65
Calcium (g)	1.25	1.58	1.00	3.72	3.30
Phosphorus (g)	1.00	0.94	0.93	2.48	1.50
Vitamin D (IU)	138.00	446.66	96.48	376.00	295.56
Supplementation					
Calcium carbonate (200 mg/day)	No	No	Yes	Yes	Yes
Calcitriol (9.84 IU/day)	No	No	No	Yes	Yes

Ingredients of the diets:

Diet 1: Dried chicken meat, tapioca, duck oil, potatoes, dried carrots, chicken oil, dried beet pulp, dried pineapple, dried citrus pulp, dried fish, salmon oil, dried duck meat, mineral salts, tomato pomace, brewer’s yeast, pea fibre, xylo-oligosaccharides, dried green tea leaves, melon juice concentrate, and milk protein powder.

Diet 2: Turkey, pork liver, chicken, pork protein isolate, rice flour, hydrolysates, fish oil, minerals, dehydrated egg whites, various sugars, vitamins, taurine, trace elements, and beta-carotene.

Diet 3: Lamb, rice, potato protein, maize, dried beet pulp, inactivated brewer’s yeast, hydrolysed animal proteins (liver), animal fat, minerals, yeast products (mannan-oligosaccharides), xylo-oligosaccharides, Yucca schidigera, algae meal, products from the processing of plants, dried garlic, glucosamine, and chondroitin sulphate.

Diet 4: Rice, turkey breast, courgettes, carrots, Parmigiano Reggiano cheese, apple, and corn oil. Supplement: maltodextrin, hydrolysed animal proteins, magnesium salts of stearic acid, corn starch, L-carnitine, calcium, group B vitamins, vitamins E, C, A, and D3, zinc, iron, and copper.

1 Minimum recommended level for adult dogs based on metabolisable energy requirements of 110 kcal/kg^0.75^ (13).

Due to personal considerations of the owners, no further treatment was implemented before day 88. At that time, although the dog’s body weight remained stable at 8.2 kg and it showed no clinical signs, blood tests revealed elevated ionised calcium values (Table 2). Therefore, a partial thyroidectomy was performed on day 90, which also resulted in the excision of the ipsilateral parathyroid glands. Histological examination of the lesion confirmed a follicular thyroid carcinoma. On day 94, the dog showed tremors, muscle spasms, and tachypnoea, caused by hypocalcaemia. This was confirmed by blood analysis (i-STAT1©, Abbott, United States), which revealed low ionised calcium at 0.84 mmol/L (RR: 1.12–1.32). The hypocalcaemia was severe enough to require admission to the ICU for continuous monitoring of vital parameters. During hospitalisation, intravenous calcium gluconate was administered (50 mg/kg over 20 min). On day 95, when the dog’s iCa reached values close to the normal range (1.35 mmol/L; RR 1.12–1.32), the infusion rate was reduced to 5 mg/kg/h for maintenance, according to the protocol suggested by de Brito Galvao et al. (9). As the patient remained clinically stable on the current treatment plan until day 97 and calcaemia returned to physiological values (1.12–1.32 mmol/L), the intravenous administration was discontinued, and oral calcium carbonate was started at a dose of 25 mg/kg/day, supplying 200 mg/day given the dog’s weight of 8.2 kg (9). However, this treatment was not sufficient to ensure long-term stabilisation of calcaemia (iCa varied from 0.61 to 0.88 mmol/L during the period from day 100 to day 124). As a consequence, on day 125, oral supplementation with calcitriol was added, supplying 9.84 IU/day (in the dosage of 30 ng/kg/day; where 1 IU = 25 ng of calcitriol) (9). Until day 93, the dog was fed D1; from day 94 to 134, the dog was fed a canned commercial diet [diet 2 (D2)] formulated for nutritional recovery during convalescence, which contained 96.48 IU/1000 kcal ME of vitamin D, 1.00 g/1000 kcal ME of calcium, and 0.93 g/1000 kcal ME of phosphorus. The maintenance energy requirement (MER) was estimated as 533 kcal ME/day (110 kcal ME/kg^0.75^) (13).

**Table 2:** Blood parameters registered during the observation period.

Parameter	Reference range	Day										
		0	88	135	156	195	225	256	282	315	350	380
iCa (mmol/L)	1.12–1.32	-	**1.65**	1.15	1.30	**1.37**	1.32	**1.36**	**1.37**	**1.35**	**1.36**	**1.38**
Total calcium (mg/dL)	7.94–12.02	**15.4**	-	8.9	11.9	9.18	8.87	11.1	9.75	9.72	9.73	11.3
Phosphate (mg/dL)	2.51–6.81	4.80	-	5.21	5.61	5.50	5.68	5.08	5.40	5.42	5.41	5.05
Magnesium (mg/dL)	1.41–2.41	-	-	1.90	1.82	1.96	1.89	2.00	1.84	1.85	1.92	1.51
Urea (mg/dL)	25–50	45	43	**78**	**72**	**73**	**86**	**111**	**107**	**77**	**73**	**117**
Creatinine (mg/dL)	<1.8	1.02	1.11	**1.90**	**2.00**	**1.95**	1.61	**2.22**	1.61	**2.08**	**2.36**	**2.06**
Triglycerides (mg/dL)	20–150	150	145	130	129	92	132	123	**153**	126	**151**	63
Cholesterol (mg/dL)	131–345	326	300	210	208	224	202	245	320	249	205	206
ALT (IU/L)	19–76	55.0	67.0	**80.0**	**98.0**	43.4	51.0	60.7	49.0	47.8	**87.0**	59.0
GGT (IU/L)	<5	4.74	**14.7**	**15.0**	**15.1**	**13.0**	**17.7**	**12.6**	**9.93**	**13.3**	**10.4**	**9.99**
ALP (IU/L)	12–134	**>1,000**	**887**	**750**	**717**	**426**	**532**	**380**	**299**	**409**	**314**	**301**
Albumin (g/dL)	2.3–3.4	3.11	-	3.20	3.34	3.30	3.05	3.26	3.23	3.19	**3.52**	3.27
Total 25(OH) vitamin D (ng/dL)	32.45–83.7	-	-	**28**	32	36	35	32	45	37	37	39

The values highlighted in bold are outside the reference ranges. iCa, total calcium, phosphate, magnesium, urea, creatinine, ALT, GGT, ALP, albumin, and total 25(OH) vitamin D reference ranges are internally determined by the laboratory; triglycerides and cholesterol reference ranges are determined according to the literature (31). iCa, ionised calcium; AST, aspartate aminotransferase; ALT, alanine aminotransferase; GGT, gamma-glutamyl transferase; ALP, alkaline phosphatase.

**Figure 2:**
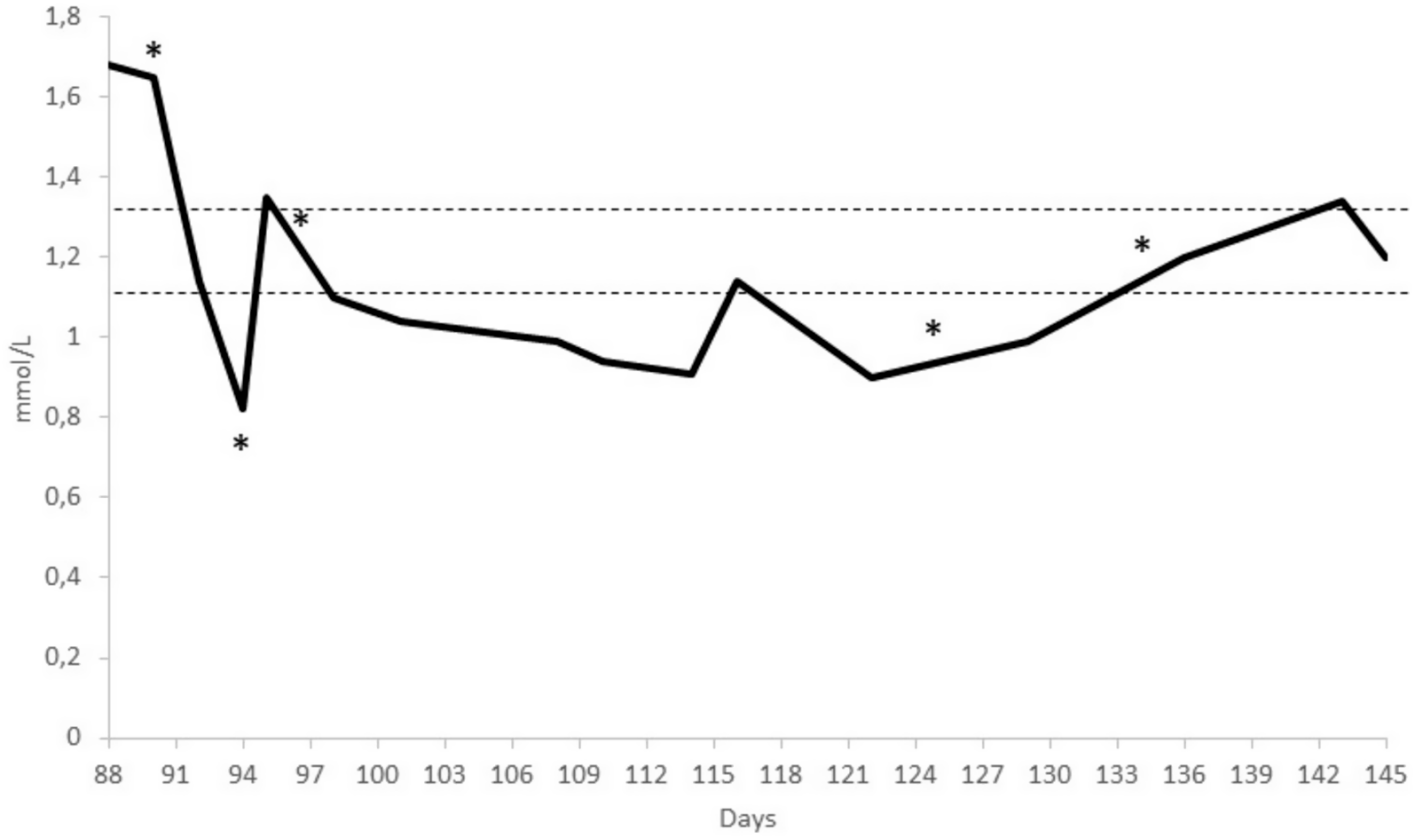
Serum ionised calcium concentration of a canine patient who underwent thyroidectomy and parathyroidectomy. The dotted lines indicate the reference interval of 1.12–1.32 mmol/L. The * highlights changes in therapeutic and dietary management: Day 90, surgery; Day 94, initiation of intravenous calcium administration and Diet 2; Day 97, cessation of intravenous calcium and transition to oral administration; Day 125, initiation of oral calcitriol therapy; and Day 135, discharge from the hospital, withdrawal of Diet 2, and introduction of Diet 3.

The dog was hospitalised for 30 days (from day 94 to 124) and then continued with day-hospital therapy for a further 21 days, during which her blood calcium levels normalised (Figure 2).

On day 135, before discharge from the VTH, a nutritional consultation was requested to provide a more appropriate diet for its conditions. The dog’s weight was 7.5 kg, its BCS was 6/9, its MCS was normal, and the MER was estimated as 499 kcal ME/day (110 kcal ME/kg^0.75^). On day 135, the energy requirements were estimated based on actual weight rather than ideal weight, taking into account weight loss during hospitalisation and possible changes in the dog’s lifestyle following discharge from the hospital. Therefore, another commercial extruded dry maintenance diet (diet 3 [D3]) was prescribed. The diet contained 3.72 g/1000 kcal ME of calcium, 2.48 g/1000 kcal ME of phosphorus, and 376 IU/1000 kcal ME of vitamin D. Calcium carbonate (200 mg) and calcitriol (9.84 IU) were maintained as part of the treatment protocol (Table 1).

### Follow-up and outcomes

From day 135 onwards, clinical follow-ups were performed monthly until day 380, involving clinical evaluations and blood sampling to monitor calcium and total 25(OH) vitamin D levels, and renal and liver biochemical biomarkers (Table 2).

On day 195, the owners reported that the dog was eating less than prescribed and that the administration of supplements had become difficult without the addition of home-cooked meat (poultry or turkey). Hyporexia was confirmed by a reduction in body weight (6.8 kg, −10%), BCS (5/9), and mild muscle loss. No other clinical signs were observed. Consequently, a specific balanced homemade diet [diet 4 (D4)] was formulated (Table 1) using software designed to meet the minimum recommended allowances as specified by FEDIAF (13). Energy requirements were calculated based on a target weight of 7 kg (110 kcal ME/kg^0.75^/day). The diet was immediately well accepted and fully consumed, providing an energy intake of 473 kcal ME/day.

On day 256, after 2 months of homemade diet administration, the dog’s weight had increased to 8 kg, BCS increased to 7/9, and its MCS had returned to normal. Consequently, the MER was reduced to 421 kcal ME/day (−11%). Trends in serum creatinine and urea concentrations (Table 2) suggested kidney damage (15). Despite the absence of polyuria and polydipsia, the urine protein-to-creatinine ratio (UPC) was monitored monthly, starting from the following month (mean UPC: 0.48 ± 0.04). Consequently, the patient was classified as International Renal Interest Society (IRIS) chronic kidney disease (CKD) stage 2, borderline proteinuric, in accordance with IRIS guidelines (15).

Once calcium levels had stabilised (Table 2), the dietary and supplementation protocols were maintained, with monthly check-ups scheduled.

## Discussion

Severe fluctuations in serum calcium levels can lead to various clinical issues, including muscle spasms, cardiac arrhythmias, ataxia, tissue calcification, and renal failure (2).

In the specific case described in this study, thyroid carcinoma caused hypercalcaemia. Surgical excision of the left thyroid lobe led to the complete removal of the left parathyroid glands, as documented previously in a similar case described by Lane and Wyatt (8). As a result, the dog developed severe hypocalcaemia, necessitating ICU admission for intravenous treatment (acute phase) and continuous monitoring of vital signs. Parenteral supplementation enables faster correction of calcaemia and reduces the risk of underdosing due to nausea or anorexia (9). After the acute phase had been resolved with a loading dose (intravenous calcium gluconate), several adaptations of the diet were necessary to stabilise serum Ca levels. Total calcium intake varied from 124 to 431 mg/kg^0.75^/day with diets D2 (from day 94 to 134) and D3 (from day 135 to 194), respectively, calculated on an ideal weight of 7 kg. After discharge from the hospital, from day 195 to day 380, the calcium intake varied from 323 to 363 mg/kg^0.75^/day with diet D4. A long period (approximately 1 year) of monitoring was necessary to achieve an acceptable serum calcium balance. Since calcium carbonate alone was insufficient, a combined treatment with calcitriol was implemented (16). This protocol effectively stabilised the patient’s serum calcium levels (Table 2). Although ionised calcium levels were slightly elevated above physiological levels between days 256 and 380, the treatment protocol was maintained. Dose adjustment was deemed impractical due to the fixed formulations of the supplements (tablets and capsules), which could have compromised owner compliance. While calcitriol monotherapy is described as a valid alternative in the literature (16), this strategy was not used in the present case. Serum total calcium and total 25(OH) vitamin D remained within physiological limits throughout the observation period. The nutritional management of the dog was changed from one diet characterised by lower calcium levels used before the surgery (1.58 g/1000 kcal ME) to one richer in this element (3.72 g/1000 kcal ME) prescribed after discharge. However, after approximately 2 months of administration, the dog showed signs of hyporexia, body weight loss, and muscle mass reduction, and a homemade diet was formulated to stimulate its appetite. A tailored homemade diet is capable of addressing the specific nutritional requirements of diseased animals (17–19). However, prioritisation of clinical conditions is necessary. The aim of the nutritional approach in this patient was to manage calcium disorders without exacerbating the underlying kidney and liver conditions. Renal calcifications, uroliths, and gallstones may be related to the hypercalcaemic (20, 21) condition present before surgery. In addition, after surgery, the dog developed hypocalcaemia, which can alter endocrine pathways and have systemic effects (2, 16). All of these conditions may be considered long-term risk factors for renal and hepatic failure, particularly in an elderly patient (5, 6, 22). In addition, patients with multiple systemic diseases often experience nausea and a reduction of food intake (23). To formulate the homemade diet, all the previously described factors were taken into consideration. Particular attention was paid to energy intake (proteins, lipids, and carbohydrates) and micronutrient balance, along with palatability. To improve palatability, ingredients that the dog had previously eaten, such as rice, turkey breast, and Parmigiano Reggiano, were chosen.

Despite alterations to renal and hepatobiliary/cholestasis blood biomarkers, no restriction to protein intake was prescribed. Indeed, the IRIS guidelines for the management of kidney disease recommend a reduction in dietary protein in the presence of proteinuria or uremic crises (15) to increase survival rates (7). However, in our case, although the UPC was indicative of borderline proteinuria, the diet D4 provided 68.73 g/1000 kcal ME, exceeding the minimum protein requirement by more than 50% (13). While this level of protein might be considered suboptimal for renal disease, this approach was chosen as inadequate protein intake appears to be a more significant risk factor than excessive intake in cases of gallbladder mucocele and cholelithiasis (24). For this diet, animal sources of protein were used, such as meat and cheese. Specific cooking instructions were provided to the owner to reduce microbial contamination and minimise the alteration of protein without compromising digestibility, e.g., cooking meat and vegetables in steam and avoiding direct exposure to heat sources (25).

The diet was formulated to ensure adequate calcium, phosphorus, and vitamin D intake, given that homemade diets are often characterised by low or imbalanced mineral contents (14). Calcium, phosphorus, vitamin D, and their interaction must be considered in the management of patients affected by different diseases, such as skeletal disorders, urolithiasis, renal failure, and gastrointestinal disorders (21).

In this specific case, calcium and calcitriol were supplemented to ensure adequate absorption and to maintain blood levels within the physiological range (9). Phosphorus is the element most strongly implicated in kidney damage, as its plasma concentration stimulates fibroblast growth factor-23 (FGF-23) (26), and the administration of a low-phosphorus diet can slow the progression of kidney disease and prolong life expectancy in dogs (7, 27). Moreover, the biological effect of phosphorus depends not only on total dietary intake but also on its chemical form and origin. Inorganic phosphorus, such as sodium or potassium phosphate salts, has greater intestinal bioavailability and a stronger phosphataemic effect compared with organic phosphorus, such as from meat or grains (7). Furthermore, some sources of phosphorus contain antinutritional factors that could negatively affect phosphorus absorption. Despite the dog’s condition of stage II CKD, no reduction in phosphorus was implemented in the dietary formulation, as the subject did not demonstrate hyperphosphataemia (15). Nevertheless, despite serum phosphate concentrations exceeding the IRIS upper limit (RR: <4.6 mg/dL) (15), they remained stable during the 8-month follow-up period. Although this stability is a positive aspect, the phosphorus content in the diet remains a limitation of the nutritional approach. Furthermore, serum phosphate may remain stable due to compensatory mechanisms. Indeed, as described in recent literature, some blood parameters, such as FGF-23 and symmetric dimethylarginine (SDMA), could be more specific indicators of renal function and hyperphosphataemia status (15, 26), but these were not evaluated in this case. Moreover, due to the therapeutic administration of calcium carbonate, the calcium-to-phosphorus ratio was slightly higher than the recommended level (13, 14) for both commercial and homemade diets. Currently, no scientific evidence exists supporting the use of such elevated calcium-to-phosphorus ratios, even in patients with chronic kidney disease. Consequently, metabolic or clinical risks may be related to the long-term use of this kind of diet. The calcium-to-phosphorus ratio is a critical determinant of mineral homeostasis and cellular physiology in dogs. Under normal conditions, phosphorus balance is tightly regulated by PTH, which promotes phosphaturia and supports normocalcaemia through its actions on the kidneys and vitamin D metabolism. However, disruption to this balance can induce pathophysiological changes, even in the absence of overt hyperphosphataemia. A high phosphorus intake leads to the suppression of renal calcitriol synthesis and reduced intestinal calcium absorption (21, 26). These alterations may not be immediately reflected in serum biochemistry. This mechanism is particularly relevant in the case under consideration, as the patient’s ability to compensate for calcium fluctuations may be compromised by postoperative hypoparathyroidism. The absence of functional parathyroid tissue may prevent the expected PTH-mediated endocrine response, thus increasing the risk of persistent disturbance to calcium homeostasis, even when serum phosphate concentrations remain within reference values.

Vitamin D exists primarily in two forms: vitamin D2 (ergocalciferol, of plant origin) and vitamin D3 (cholecalciferol, of animal origin). Dogs are capable of utilising both dietary forms with equal efficiency (28). Following intestinal absorption, vitamins D2 and D3 are metabolised into various intermediates, culminating in the synthesis of calcitriol (1). In the literature, a relationship between vitamin D supplementation and canine gallbladder mucocele has been suggested (29, 30); specifically, an excessive ergocalciferol administration has been linked to high bile calcium concentration (30). In this case, it was necessary to add an adequate amount of vitamin D to the diet to finally stabilise blood Ca values.

Although the dog showed sporadic increases in triglyceride values (day 282 and day 350, Table 2), these values were not considered to be indicative of hyperlipidaemia, in accordance with the American College of Internal Medicine (ACVIM) guidelines (31). Therefore, no specific nutritional restriction of fats was prioritised in the dietary plan.

Alkaline phosphatase levels were very high, ranging from over 1,000 IU/L on day 0 to 301 IU/L on day 380 (Table 2). Elevated ALP levels can indicate a wide range of diseases (32). This is due to different isoenzymes, which are specific to different organs. In particular, three types of isoenzymes are clinically relevant in dogs: biliary, bone, and cortisol-induced (32). It is not possible to distinguish between these types using routine laboratory tests. Therefore, the cause of the elevated ALP levels in this dog remains unclear. However, as the dog’s ALP levels decreased drastically during the observation period, this was not taken into account when planning its diet. In addition, the high levels of gamma-glutamyl transferase (GGT) observed during the entire observation period could be suggestive of biliary suffering.

Careful nutritional planning was essential during the follow-up period in this case, particularly in view of the prolonged calcium carbonate supplementation and changes in dietary composition, to avoid exacerbation of the calcium–phosphorus imbalance and its potential long-term effects on renal function. However, further assessments, such as monitoring for lithiasis and renal and hepatic function, may require modification of supplement dosages.

## Conclusion

In the present case, hypocalcaemia in a dog undergoing thyroidectomy and parathyroidectomy was corrected and subsequently maintained within a slightly hypercalcaemic range through the daily administration of 323–431 mg/kg^0.75^ of calcium, 29.7– 43.5 IU/kg^0.75^ of vitamin D, and 139– 273 mg/kg^0.75^ of phosphorus, supplemented with calcitriol at a dose of 9.84 IU/day.

## Data Availability

The original contributions presented in the study are included in the article/supplementary material; further inquiries can be directed to the corresponding author.
